# Feasibility of Intraoperative Tissue Oxygen Saturation Imaging Using OXEI Technology During Robotic Esophagectomy: A Case Series

**DOI:** 10.1177/15569845251401204

**Published:** 2026-02-03

**Authors:** Susana Fortich, Jennifer Den, Mathew Thomas, Roman Petrov

**Affiliations:** 1 Department of Surgery, University of Texas Medical Branch, Galveston, TX, USA; 2 Division of Cardiovascular and Thoracic Surgery, Department of Surgery, Mayo Clinic Florida, Jacksonville, FL, USA; 3 Division of Cardiovascular and Thoracic Surgery, Department of Surgery, University of Texas Medical Branch, Galveston, TX, USA

**Keywords:** tissue perfusion, esophagectomy, ICG, robotic surgery

## Abstract

**Objective:**

Esophageal cancer is a leading cause of cancer-related mortality, with a 5-year survival rate of 20%. Surgical resection remains the primary treatment for early and locally advanced disease. Anastomotic leak is a major concern, which significantly increases morbidity and mortality. Impaired conduit perfusion and tissue ischemia are key risk factors. This series describes the use of ELUXEO Oxygen Saturation Endoscopic Imaging (OXEI) technology (Fujifilm Healthcare Americas Corp, Lexington, MA, USA) to assess tissue oxygen saturation during esophagectomy.

**Methods:**

OXEI was used in 6 cases to evaluate conduit perfusion during esophagectomy procedures. Real-time hemoglobin oxygen saturation imaging identified ischemic areas, with StO_2_ levels ranging from 17% in poorly perfused regions to 92% in well-perfused areas. OXEI findings were congruent with indocyanine green fluorescence imaging but avoided dye-related limitations.

**Results:**

OXEI technology offered a dye-free alternative that allowed real-time assessment of tissue oxygenation, facilitating accurate perfusion quantification. Its reproducibility without dye administration and limitations of tissue saturation or washout concerns added reliability, especially during long multistage procedures. In addition, OXEI has been shown to be consistent irrespective of distances from the targeted area, providing precise tissue saturation quantification throughout critical steps of esophagectomy. These findings highlight the potential of this technology as a valuable adjunct in esophageal surgery.

**Conclusions:**

The experience with ELUXEO technology in esophagectomy is promising. It provides a reliable, dye-free method for real-time perfusion assessment, potentially reducing the incidence of anastomotic leaks, preventing dye-associated complications, and improving surgical outcomes. Further studies are warranted to validate these findings in esophagectomies.

## Graphical abstract

**Visual abstract:**
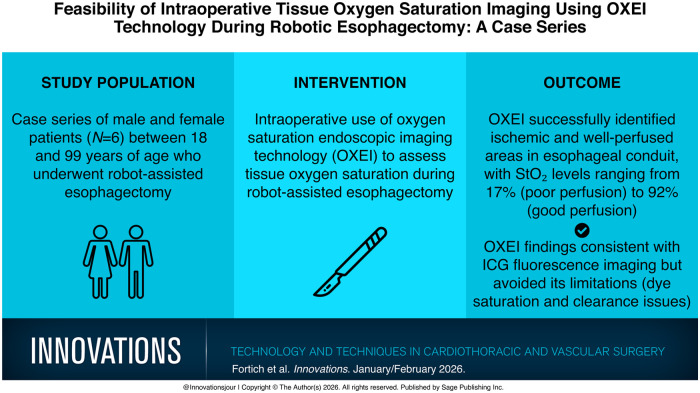

**Central Message**

OXEI technology provides real-time, dye-free evaluation of hemoglobin tissue oxygen saturation, offering a potential superior alternative to indocyanine green fluorescence imaging. This study presents our experience using OXEI to assess tissue perfusion during robotic esophagectomy. The findings suggest OXEI may improve intraoperative decision-making and reduce complications.

## Introduction

Esophageal carcinoma is the sixth leading cause of cancer mortality worldwide.^1^ Surgical resection remains the mainstay treatment for early-stage and locally advanced esophageal cancer. Esophagectomy is a complex oncologic and reconstructive procedure with a high rate of postoperative morbidity even in the best hands and high-volume centers. Anastomotic leak is one of the most common and severe complications of esophagectomy and is associated with increased postoperative mortality, reaching up to 60%, as well as increased morbidity, particularly prolonged hospital stay, increased cost of care, increased reintervention rate, increased cancer recurrence rates, decreased overall survival, increased rate of anastomotic strictures, postoperative dysphagia, and poorer quality of life.^2,3^

Multiple factors are implicated as the risks of anastomotic leak, including the patient’s nutritional status and comorbidities, neoadjuvant therapy, details of surgical technique, and anastomotic location. Poor tissue perfusion and the conduit ischemia are major risk factors in the development of anastomotic leaks.^4^ Indocyanine green (ICG) video angiography has been introduced into clinical practice for qualitative assessment of the microcirculation and tissue perfusion at the anastomotic site. However, although rare, the administration of ICG is not free from risk, and adverse events range from mild to severe.^5^ Technologies such as oxygen saturation endoscopic imaging (OXEI) now allow direct assessment of tissue oxygen saturation and present new ways to mitigate this risk. The goal of this study is to describe the use of new tissue oxygen saturation imaging in the assessment of conduit ischemia in patients undergoing esophagectomy.

## Methods

### Technical Aspect of the Technology Application

OXEI is a passive, dye-free imaging modality that does not require intravenous dye administration. It leverages multispectral visible light to assess relative levels of tissue oxygenation through differential light absorption by oxygenated and deoxygenated hemoglobin. Using multispectral illumination, the technology provides a visual representation of the levels of tissue hemoglobin saturation. Oxy-hemoglobin and deoxy-hemoglobin have different absorption properties in the visible light wavelength range. The distribution of tissue hemoglobin oxygen saturation (StO_2_) levels is measured and projected into a real-time image using multispectral illuminations. This is done utilizing unique 5-color (violet, blue, green, amber, red) LED endoscopic imaging technology that evaluates StO_2_ levels. The image has 2 modes: a heatmap mode and an overlay mode. In heatmap mode, the rainbow color panel represents different hemoglobin oxygen saturation levels, with bright red being equivalent to 100%, yellow being equivalent to 60%, and dark blue being equivalent to 0%. This technology is Food and Drug Administration approved for gastrointestinal resection, endoscopy, and bariatric procedures since 2020.

**Fig. 1.**
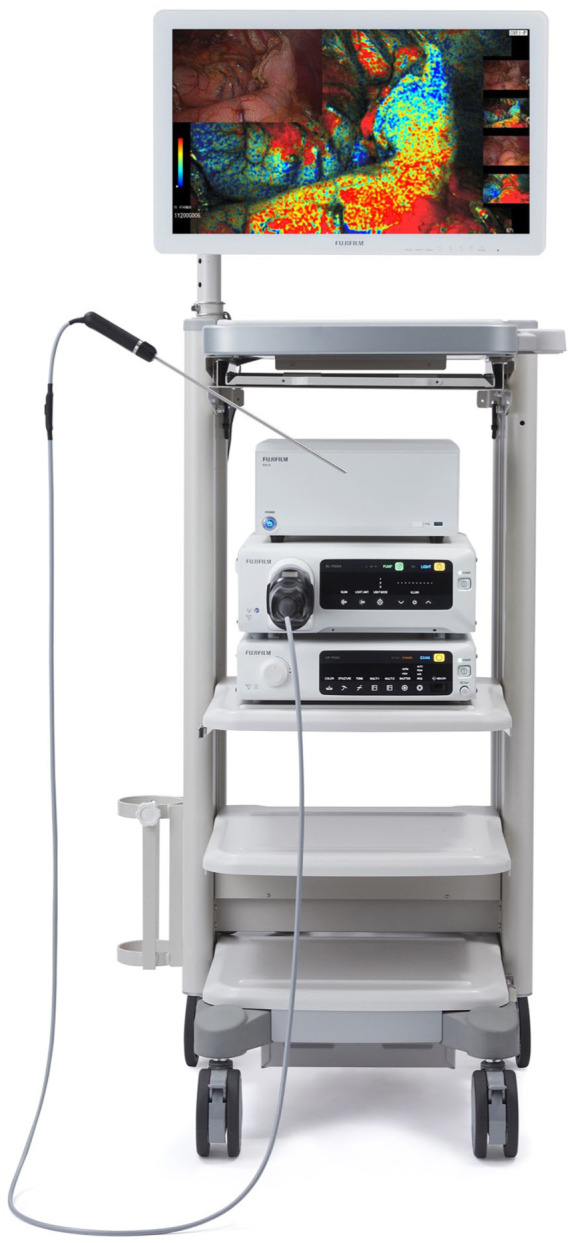
The FUJIFILM ELUXEO Vision system features dye-free, real-time tissue oxygen saturation imaging and is compatible with select FUJIFILM laparoscopes and endoscopes. It allows multiple StO_2_ assessments on both serosal and mucosal tissues of the gastrointestinal tract during procedures without time restrictions. Images courtesy of FUJIFILM Corporation. Reproduced with permission.

The ELUXEO Vision cart (Fujifilm® Healthcare Americas Corp, Lexington, MA, USA) was used in this study. ELUXEO technology is incorporated in the manufacturer’s tower and requires an external connection to a robotic vision cart for image delivery (Fig. 1). The image is delivered into the screen on the surgeon’s console with “picture-in-picture” mode. Notably, the robotic light source interferes with tissue oxygen saturation mapping and must be turned off. The robotic camera removal leads to locking of the working instruments and loss of control. As such, the robotic light source is turned off with the camera in place. Importantly, this can be accomplished only at the vision cart. The laparoscopic Fuji camera is advanced through the assistant port and allows surgical field illumination and assessment for tissue oxygenation and the microcirculation.

### Patient Position and Port Placement

All procedures were performed on the da Vinci Xi surgical platform (Intuitive, Sunnyvale, CA, USA). For the abdominal part of the esophagectomy, standard port placement was used, with all 4 ports placed horizontally one-third of the distance between the umbilicus and the xiphoid process, as previously reported.^6,7^ A 12 mm assistant port was placed at the level of the umbilicus between the second and third arms. Regardless of the type of esophagectomy performed, the standard abdominal part procedure included on-table endoscopy with submucosal injection of 200 units of Botox into the pylorus, followed by intra-abdominal part with hiatal dissection, Kocher maneuver, division of the left gastric artery at the takeoff from the celiac trunk with lymphadenectomy in oncologic cases, mobilization of the greater gastric curvature with preservation of the right gastroepiploic artery, and creation of the narrow (5 cm) gastric conduit by serial stapling from the incisura along the greater curvature. The omental flap was harvested and based on the short gastric vessels at the mid-gastric body. After completion of the conduit preparation, an assessment of tissue oxygenation was performed using ELUXEO technology, and quantifiable results were presented. Following this step, the Fujifilm camera was withdrawn, and a standard assessment of the microcirculation with ICG was performed. The robotic camera was turned on, and Firefly mode was activated. Three milliliters of ICG (7.5 mg) was administered intravenously. For the adequacy of the perfusion, 25 s from the celiac artery to the conduit tip tissue ICG saturation was used.

Based on the type of esophagectomy, variations occurred in the thoracic or cervical part of the procedure. In McKeown esophagectomy, the thoracic portion was performed first, followed by the abdominal and cervical steps of the procedure. In Ivor Lewis esophagectomy, the abdominal portion was performed first, followed by the thoracic stage. In robotic transhiatal esophagectomy, all intrathoracic dissection was performed during the abdominal part of the procedure. Repeat assessment of the conduit with ICG prior to performance of the anastomosis was ineffective in transhiatal and McKeown esophagectomy, as the tissue remained saturated with dye due to a short interval between steps. It was variable in Ivor Lewis esophagectomy due to longer washout time, and in 1 case could be employed again. However, regardless of the approach, tissue oxygen saturation was successfully performed both in the abdomen and in either the chest or neck prior to anastomosis.

## Results

### Case 1

**Fig. 2.**
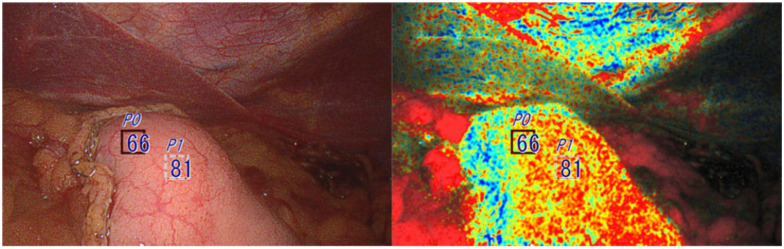
Oxygen saturation endoscopic imaging was used only once right after creating a gastric conduit inside the abdominal cavity. The conduit was not additionally resected prior to anastomosis. The StO_2_ levels within the squares are 66% and 81%.

A 65-year-old male patient with recurrent esophageal adenocarcinoma underwent a salvage robotic McKeown esophagectomy 15 months after definitive chemoradiation therapy. OXEI was applied intraabdominally after gastric conduit creation, showing excellent perfusion of the conduit tip at 81% StO_2_ (Fig. 2). This finding corroborated with ICG fluorescence assessment, demonstrating good perfusion. However, OXEI identified borderline tip ischemia after conduit delivery into the cervicotomy wound prior to anastomosis. ICG did not capture this due to tissue dye saturation showing full conduit fluorescence. The finding of tip ischemia led to modification of the surgical technique with additional mobilization and further conduit transposition and tip resection. Postoperatively, the patient developed anastomotic leak on postoperative day 7, treated with cervicotomy opening, external drainage, and local wound care, eventually leading to the closure of the leak within 2 weeks of the original procedure without additional complications. This was the only procedure complicated by the development of anastomotic leak in our series. This case highlights the high-risk nature of salvage intervention as a potential risk factor for anastomotic complications, even in the setting of imaging-confirmed perfusion, and warrants further study.

### Case 2

**Fig. 3.**
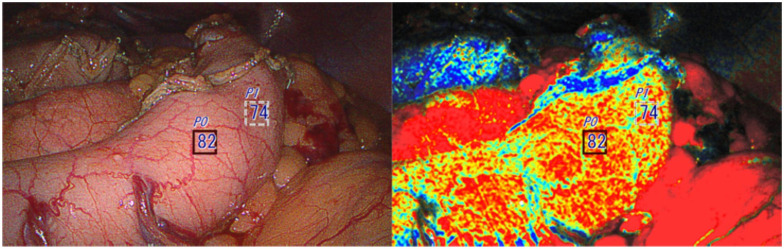
The left image shows the first observation right after creating a gastric conduit inside the abdominal cavity. The conduit was not additionally resected.

A 63-year-old male patient with a history of stage IIIB (T3,N1,M0) esophageal adenocarcinoma underwent robotic Ivor Lewis esophagectomy after undergoing induction chemoradiation therapy. OXEI revealed conduit tissue saturation with StO_2_ levels between 82% and 74%, corresponding with good perfusion on ICG videoangiography. After delivery of the conduit into the chest, a repeat oxygen saturation assessment confirmed excellent perfusion, corroborating ICG finding after repeat dye administration after complete washout (Fig. 3). The postoperative course was uneventful, without the development of an anastomotic leak.

### Case 3

**Fig. 4.**
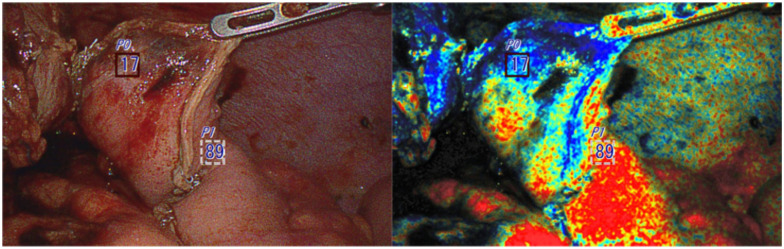
The first observation right after creating a gastric conduit inside the abdominal cavity. The tip of the conduit (less oxygenated area) was additionally resected prior to anastomosis.

A 42-year-old female patient with a history of end-stage achalasia and previous Heller myotomy, progressive dysphagia, and failure to thrive underwent robotic transhiatal esophagectomy. During the preparation of the gastric conduit, OXEI detected low StO_2_ (17%) at the conduit tip (Fig. 4), concurrent with ICG fluorescence findings, demonstrating poor perfusion. After conduit transposition into the neck, similar findings on the oxygen saturation imaging were confirmed, discordant from ICG fluorescence imaging, due to tissue dye saturation. Additional mobilization of the conduit was performed, and the anastomosis was positioned at the well-perfused part of the conduit, excluding and resecting the ischemic tip. The patient had an uneventful postoperative course with no evidence of an anastomotic leak.

### Case 4

**Fig. 5.**
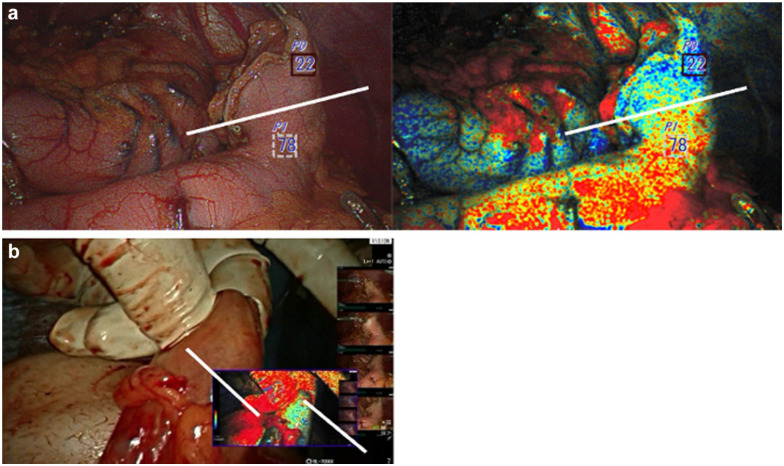
Oxygen saturation endoscopic imaging. (a) Image shows the first observation right after creating a gastric conduit inside the abdominal cavity. (b) Image shows the second observation in the cervicotomy wound. The tip of the conduit (less oxygenated area) identified in the first and second observations was additionally resected prior to anastomosis.

A 45-year-old male patient with poorly differentiated stage III (T2N1M0) esophageal adenocarcinoma, underwent robotic Ivor Lewis esophagectomy after undergoing induction chemoradiation therapy. OXEI showed good perfusion throughout the procedure, concordant with ICG fluorescence findings. The case was converted to an open thoracotomy due to complete pleural space obliteration by adhesions. During anastomosis creation inside the chest, the tip of the conduit was poorly perfused at 22% (Fig. 5). ICG fluorescence was unreliable due to tissue saturation and full conduit fluorescence. The ischemic tip of the conduit was resected, and the anastomosis was placed at the level of well-perfused tissue. The postoperative course was uneventful, and there was no occurrence of anastomotic leak.

### Case 5

**Fig. 6.**
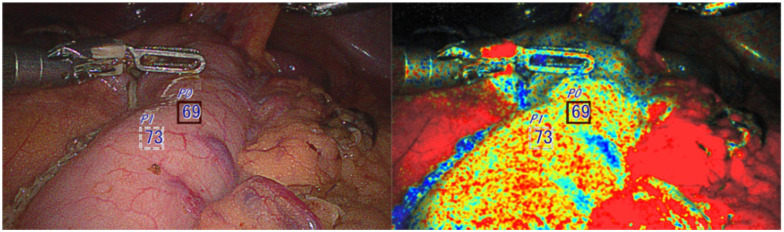
Good saturation of the conduit confirmed by the oxygen saturation endoscopic imaging technology.

A 76-year-old female patient underwent robotic Ivor Lewis esophagectomy after induction treatment for stage cT2N1 squamous cell carcinoma of the gastroesophageal junction in the settings of achalasia and a previous peroral endoscopic myotomy procedure. The specimen confirmed complete pathologic response (ypT0N0). Intraoperatively, OXEI demonstrated good saturation of the conduit (Fig. 6), corroborated with the ICG imaging in the peritoneal cavity. After conduit delivery into the chest prior to anastomosis, oxygen saturation remained high, and ICG imaging was unusable due to tissue saturation and full conduit fluorescence. Standard circular mechanical anastomosis was created. The postoperative course was uncomplicated with excellent healing without a leak.

### Case 6

**Fig. 7.**
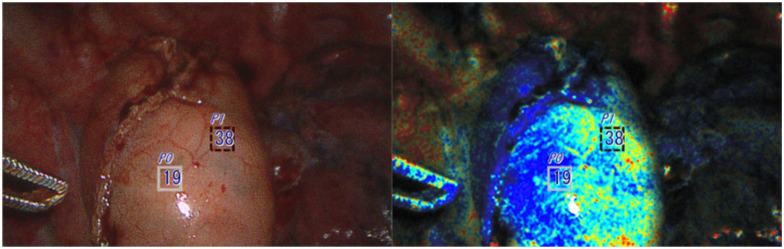
The ischemic tip of the conduit (less oxygenated area) identified using the oxygen saturation endoscopic imaging technology was additionally resected prior to anastomosis.

A 52-year-old male patient with stage II (T2, N0, M0) esophageal adenocarcinoma had a robot-assisted Ivor Lewis esophagectomy after undergoing induction chemoradiation therapy. OXEI showed good perfusion throughout the procedure, findings concordant with ICG fluorescence. During the intrathoracic anastomosis, the tip of the conduit was poorly perfused at 19% (Fig. 7). ICG fluorescence was unusable due to tissue saturation. The ischemic tip of the conduit was resected with anastomosis application at the well-saturated level. All margins were negative for invasive carcinoma. The postoperative course was uncomplicated without the appearance of a leak.

## Discussion

Anastomotic leak and the conduit necrosis are the most feared and severe complications following esophagectomy. The rate of anastomotic leaks varies in the available literature owing to differences in surgical technique, type of procedure, center experience, and age of the publication.^8–11^

Recently, with the advent of robotic technology, the use of ICG fluorescence has enjoyed wide adoption. However, the results of its application are contradictory. In a meta-analysis by Ladak et al., the implementation of ICG fluorescence led to a decrease in the rate of anastomotic leaks by about 70%.^12^ On the contrary, a study of 181 patients conducted by Banks et al. showed that ICG use was associated with an increased leak rate and higher mortality, although additional findings suggested that abnormal intraoperative ICG was predictive of postoperative outcomes.^13^ Another retrospective cohort study involving 312 patients by LeBlanc et al. showed no significant difference in the anastomotic leak rate with or without ICG use.^14^ Notably, a discordance between surgeon-observed and ICG-augmented line of demarcation was seen in 80% of cases.

One of the main drawbacks of ICG is a lack of standardization. There is no established quantitative threshold of fluorescence signal to determine the adequacy of the tissue perfusion. Furthermore, there is no universally accepted dosage of ICG, ranging from 1.25 to 25 mg per bolus. A lower dose may not be enough to provide a clear assessment, and a higher dose may interfere with a subsequent measurement if the background signal remains high due to tissue saturation and impaired clearance of the dye from the blood flow.^15^ Notably, although rare, severe complications of ICG administration have been described in the literature, particularly anaphylactic reaction.^16^

Thus, our case series describes Fujifilm’s ELUXEO as another promising method with superior features. This technology enables real-time visualization of tissue oxygen saturation by taking advantage of hemoglobin’s optical properties with the ability of quantifiable assessment. This technology does not require the use of proxy media, such as fluorescent dye to indirectly extrapolate oxygen delivery from a flow rate. The system-generated image provides precise delineation of tissue oxygen saturation and allows the surgeon to select an ideal site for anastomosis placement for improved anastomotic viability.

Our case series used both methods of anastomotic perfusion assessments during robot-assisted minimally invasive esophagectomies as a proof-of-concept experience. In our limited initial series (Table 1), there was an excellent concordance of the tissue oxygenation and ICG fluorescence qualitative data on the initial imaging after conduit creation. Tissue oxygenation imaging remained a viable option throughout continuity of the procedure, providing valuable diagnostic information at each step of the esophagectomy. In our experience, due to tissue saturation with ICG and lack of washout in some cases, ICG fluorescence was usable only during the initial assessment after conduit preparation.

**Table 1.** Patient Characteristics.

Characteristic	Case 1	Case 2	Case 3	Case 4	Case 5	Case 6
Age, years	65	63	42	45	76	52
Sex	Male	Male	Female	Male	Female	Male
Race	Hispanic or Latino	White	White	White	White	White
BMI, kg/m^2^	24.6	24.7	22.7	24	24.4	20.1
Diagnosis	Esophageal adenocarcinoma	Esophageal adenocarcinoma	End stage achalasia	Esophageal adenocarcinoma	Esophageal squamous cell carcinoma	Esophageal adenocarcinoma
Stage (c)	Stage IVA (T4, N3, M0)	Stage III (T3, N1, M0)	—	Stage III (T2, N1, M0)	Stage II (T2, N1, M0)	Stage II (T2, N0, M0)
Induction treatment	Carboplatin, paclitaxel, IMRT	Carboplatin, paclitaxel, IMRT	—	Carboplatin, paclitaxel, IMRT	Carboplatin, paclitaxel, IMRT	Fluorouracil, leucovorin, oxaliplatin, docetaxel, IMRT
Type of surgery	Robotic McKeown esophagectomy	Robotic Ivor Lewis esophagectomy	Robotic transhiatal esophagectomy	Robotic Ivor Lewis esophagectomy	Robotic Ivor Lewis esophagectomy	Robotic Ivor Lewis esophagectomy

Abbreviations: BMI, body mass index; C, clinical; IMRT, intensity-modulated radiation therapy.

## Conclusions

Our case series is the first esophagectomy series using the Fujifilm ELUXEO technology to evaluate conduit oxygen saturation, with promising results. ICG perfusion assessment is a useful technique for tissue perfusion assessment, but the Fujifilm ELUXEO technology offers several advantages. These include real-time visualization of tissue oxygen saturation without need for injectable dyes, completely avoiding the risk of potential complications. The technology allows for continuous, repeatable measurements without the limitations of dye saturation, and it is effective for both laparoscopic and endoscopic visualization. Given these benefits, ELUXEO provides valuable feedback to identify ischemic tissue when ICG fluorescence is limited, allowing a tailored approach to the conduit site selection.
